# Measurement of myocardial blood flow in atrial fibrillation using high-resolution, free-breathing in-line quantitative cardiovascular magnetic resonance

**DOI:** 10.1016/j.jocmr.2025.101917

**Published:** 2025-06-06

**Authors:** Richard J. Crawley, Karl-Philipp Kunze, Anmol Kaushal, Xenios Milidonis, Jack Highton, Blanca Domenech-Ximenos, Irum D. Kotadia, Can Karamanli, Nathan C.K. Wong, Robbie Murphy, Ebraham Alskaf, Radhouene Neji, Mark O’Neill, Steven E. Williams, Cian M. Scannell, Sven Plein, Amedeo Chiribiri

**Affiliations:** a School of Biomedical Engineering & Imaging Sciences, King’s College London, London, United Kingdom; b Guy’s and St Thomas’ NHS Foundation Trust, London, United Kingdom; c Magnetic Resonance Research Collaborations, Siemens Healthcare Limited, Camberley, United Kingdom; d DeepCamera MRG, CYENS Centre of Excellence, Nicosia, Cyprus; e Department of Medical Physics and Biomedical Engineering, University College London, London, United Kingdom; f Department of Radiology, Hospital Clínic de Barcelona, Barcelona, Spain; g Centre for Cardiovascular Science, University of Edinburgh, Edinburgh, United Kingdom; h Department of Biomedical Engineering, Eindhoven University of Technology, Eindhoven, the Netherlands; i Leeds Institute of Cardiovascular and Metabolic Medicine, University of Leeds, Leeds, United Kingdom

**Keywords:** Perfusion, Quantitative, Atrial fibrillation, MBF

## Abstract

**Background:**

Stress perfusion cardiovascular magnetic resonance (CMR) in the presence of atrial fibrillation (AF) has long been challenging due to electrocardiogram (ECG) mis-triggering. However, non-invasive ischemia imaging is important due to an increased risk of myocardial infarction in patients with AF, which has been attributed to underlying microvascular dysfunction. Myocardial blood flow (MBF) in patients with AF is poorly understood, and few studies have attempted to quantify this through non-invasive imaging.

**Methods:**

Patients were recruited for stress perfusion CMR using a research sequence at 3-Tesla. Image acquisition occurred during both vasodilator-induced hyperemia and at rest. Stress and rest MBF maps were automatically generated. Analysis of perfusion maps included assessment of myocardial perfusion reserve (MPR) and endocardial-to-epicardial MBF ratios.

**Results:**

Around 442 patients were analyzed; 63 of whom had a history of AF and were in AF during the scan. Both MBF during hyperemia (stress MBF) and MPR were reduced in patients with AF compared to those in sinus rhythm (median stress MBF 1.85 [1.52–2.24] vs. 2.35 [1.98–2.77] mL/min/g, p<0.001; median MPR 1.95 [1.62–2.19] vs. 2.37 [2.05–2.80], p<0.001). No significant difference was seen between the two groups at rest (p=0.451). When considering co-factors affecting MBF, multivariate linear regression analysis identified the presence of AF as a significant independent contributor to stress MBF and MPR values. Both endocardial and epicardial stress MBF and MPR were reduced in AF compared with sinus rhythm (both p<0.001) and endocardial/epicardial ratios were similar between the groups.

**Conclusion:**

Automated quantitative MBF assessment can be performed in patients with AF. At hyperemia, MBF is reduced in AF compared to sinus rhythm.

## Graphical abstract

**Figure:**
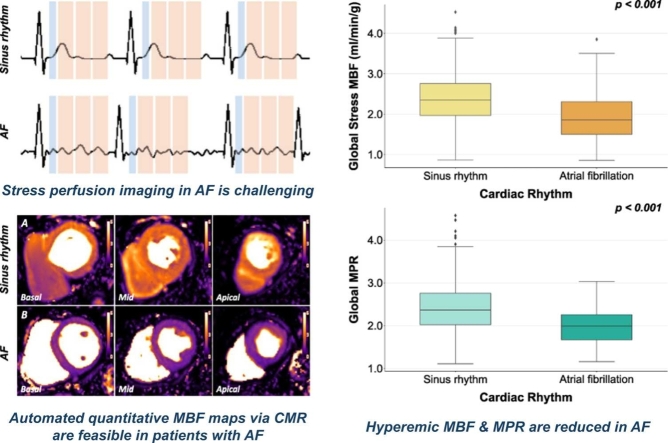
Assessment of hyperaemic MBF in patients with AF is feasible using high-resolution, free-breathing stress perfusion cardiovascular magnetic resonance with automated in-line quantitative mapping. Hyperaemic MBF and MPR are both reduced in patients with AF compared to those in sinus rhythm. *MBF* myocardial blood flow, *AF* atrial fibrillation, *MPR* myocardial perfusion reserve.

## 1 Introduction

The global incidence of atrial fibrillation (AF) has increased over the last two decades and presents a major challenge for healthcare systems [1]. With exertional symptoms similar to angina, patients are often investigated for concomitant coronary artery disease (CAD) [2]. Indeed, there appears to be an association between AF and myocardial infarction (MI), even in the absence of CAD and cardiovascular risk factors [3]. Whilst there is an increased risk of thromboembolic MIs in those with AF, rates of non-ST-elevation MI are also higher, suggesting that there may be a reduction of myocardial blood flow (MBF) in AF [4], [5]. Previous studies attempting to assess MBF in patients with AF demonstrated lower hyperemic MBF in the presence of AF compared with patients in sinus rhythm [6], [7], [8], [9], [10]. Beat-to-beat irregularity and changes in cardiac output may contribute to reduced hyperemic MBF, but it has been suggested that other mechanisms, such as oxygen supply-demand mismatch, may also have a significant effect [8]. Coronary microvascular/endothelial dysfunction and increased oxidative stress have been observed in patients with longstanding AF and may contribute to reduced MBF during hyperemia [8], [11], [12].

Stress perfusion CMR has become more widely available in recent years, and is recommended as a first-line functional investigation for CAD [13]. Irregularity in electrocardiogram (ECG)-gating seen in AF makes stress perfusion CMR in these patients technically challenging, with risk of both image artifact and reduction in temporal resolution due to sequence mis-triggering. Despite these challenges, stress perfusion CMR in patients with AF is feasible, and visual analysis demonstrates good diagnostic performance and prognostic value [14], [15].

The development of quantitative stress perfusion CMR methods has allowed the accurate detection of CAD by assessing MBF both at rest and during vasodilator-induced hyperemia [16], [17], [18]. However, there is a sparsity of research applying quantitative stress perfusion CMR analysis in patients with AF. A single-center study demonstrated reduced MBF in a small number of patients with persistent AF compared with matched controls in sinus rhythm, but no other studies have attempted to assess this apparent difference in MBF between AF and sinus rhythm [6]. The development of automated quantitative perfusion CMR sequences now allows the assessment of these patients in larger numbers [19], [20].

The goal of this study was to assess MBF in patients with AF using a high-resolution free-breathing perfusion CMR sequence with automated in-line quantitative MBF mapping [18]. The specific aims of the study were first, to assess whether quantitative MBF assessment was feasible in patients with AF; and second, to assess the differences in MBF between AF and sinus rhythm and determine factors that contribute to this.

## 2 Methods

### 2.1 Study population

Patients aged 18 to 90 were prospectively recruited at St Thomas’ Hospital, London, United Kingdom between January 2022 and May 2023. All patients had been clinically referred for investigation by stress perfusion CMR due to symptoms suggestive of CAD. Patients were recruited to one/both of two ongoing large cohort studies, providing written informed consent on the day of their scan, with both studies having received ethical approval from UK Research Ethics Committees (ref 15/NS/0030, North of Scotland; and 22/NW/0309, North West – Haydock).

The participants underwent stress perfusion CMR using a research sequence deployed within the scanner’s native software framework, capable of producing high-resolution perfusion maps visible to the scan operators within 2–3 min [21]. Cardiac rhythm assessment was typically conducted by the supervising clinician using in-scanner ECG monitoring equipment. The participant's underlying rhythm was classified as either sinus rhythm or atrial fibrillation—other cardiac rhythms were not observed. Participants classified as having AF were known to have a history of persistent or permanent AF. Any patients with a history of paroxysmal AF, atrial arrhythmia, or treated AF (by direct current cardioversion or catheter ablation) were excluded from further analysis due to the potential for bias. Participants with a resting heart rate (HR) greater than 100 beats per minute (bpm) were not recruited due to the potential for ECG mis-triggering. Patients were also excluded if there was evidence regional or circumferential inducible hypoperfusion or systemic/pulmonary fluid congestion requiring urgent therapeutic intervention.

### 2.2 Image acquisition

All study participants underwent stress perfusion CMR on a single 3-Tesla scanner (MAGNETOM Vida, Siemens Healthineers AG, Erlangen, Germany) with an 18-channel flexible phased array coil and 72-channel spine coil array. All patients were asked to abstain from nicotine and caffeinated products for 24 h prior to the CMR. Perfusion imaging was conducted without breath-hold (i.e., free-breathing) and consisted of 60–100 dynamic acquisitions using a saturation recovery fast gradient echo research sequence. 0.075 mmol/kg gadobutrol (Gadovist, Bayer AG, Leverkusen, Germany) was administered at a rate of 4 mL/s with 20 mL saline flush during each acquisition. Image acquisition included three high-resolution short-axis slices of the left ventricle during each R-R interval using a modified temporally incoherent k-space sampling pattern with an acceleration factor of 5 [21]. Within the same image acquisition, a dual-sequence acquisition of a low-resolution arterial input function (AIF) slice was obtained at the basal ventricular level. Typical imaging parameters for the perfusion sequence are described in previous studies [18], [22].

Image acquisition was conducted during vasodilator-mediated hyperemia, and then again at rest (≥10 min following stress imaging). One of either adenosine or regadenoson was used to induce hyperemia, with the most appropriate agent selected by the supervising clinician according to a local safety assessment. Adenosine was administered as a continuous intravenous infusion, with a starting dose of 140 μg/kg/min. If no response was seen within 2 min, the dose was up-titrated (increasing to 175 μg/kg/min, then 210 μg/kg/min if required) every 1–2 min to ensure appropriate increase in patient HR, change in systolic and diastolic BP, and/or symptomatic response. Image acquisition occurred at 3–6 min after commencement of the infusion. Regadenoson was administered as a single 400 μg intravenous bolus with 10 mL saline flush, with imaging typically conducted at 2 min after administration. Haemodynamic measurements (blood pressure [BP] and HR) were conducted at baseline and peak hyperemia for all patients. Mean arterial pressure (MAP) was calculated as diastolic BP plus ⅓ (systolic BP minus diastolic BP). Rate-pressure product (RPP) was calculated as HR multiplied by systolic BP.

### 2.3 Image reconstruction

**Fig. 1:**
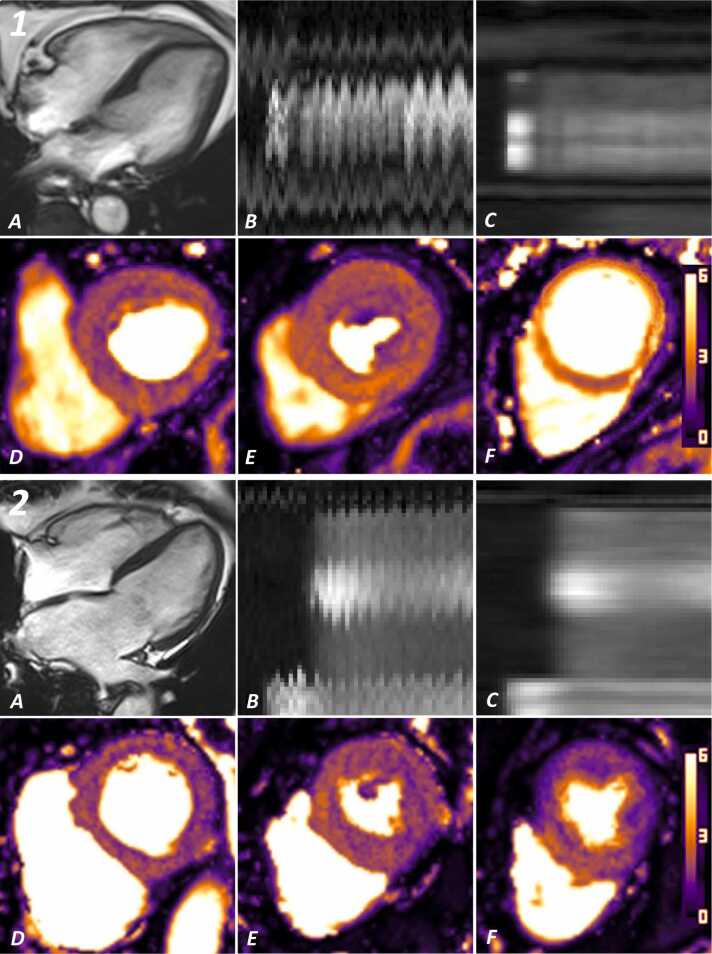
Example images acquired from two similar patients: patient 1 is in sinus rhythm at time of scanning while patient 2 is in atrial fibrillation. Both patients are aged 75 and female, with LVEF > 60% and no ventricular late-gadolinium enhancement. For each patient: (A) four-chamber cine in end-diastole; (B) motion profile image of the perfusion sequence (mid ventricular slice); (C) motion profile image of the perfusion sequence (mid ventricular slice) after the motion-compensation has been applied; (D, E & F) in-line quantitative perfusion maps (basal, mid and apical respectively) acquired during adenosine administration. Patient 1: global stress MBF 2.36 mL/min/g. Patient 2: global stress MBF 1.89 mL/min/g. Perfusion sequence cines for each patient are demonstrated in the supplementary videos. *LVEF* left ventricular ejection fraction, *MBF* myocardial blood flow

All image reconstruction and perfusion map generation were conducted in-line on the scanner. The reconstruction pipeline featured an iterative framework with integrated consecutive-image motion compensation and temporal regularization, and has been described previously [18], [21], [22], [23]. MBF values were calculated using first-pass Fermi-constrained deconvolution applied to the high-resolution myocardial series [24]. All perfusion images and quantitative maps were visible on the scanner console within 2–3 min. Fig. 1 demonstrates an example of the in-line perfusion maps during hyperemia for both sinus rhythm [Patient 1] and atrial fibrillation [Patient 2].

Supplementary material related to this article can be found online at doi:10.1016/j.jocmr.2025.101917.

The following is the Supplementary material related to this article Video S1,Video S2..

Video S1. Mid-ventricular slice of Patient 1 (see Fig. 1) who was in sinus rhythm during hyperemic acquisition. The left pane represents the low-resolution series acquired. The right pane demonstrates the high-resolution series with application of the motion-compensation framework

Video S2. Mid-ventricular slice of Patient 2 (see Fig. 1) who was in atrial fibrillation during hyperemic acquiisition. The left pane represents the low-resolution series. The right pane demonstrates the high-resolution series with application of the motion-compensation framework

### 2.4 Image and perfusion map analysis

Scans from all patients were visually evaluated by two fully accredited physicians with greater than 3 years of CMR experience. Scans were assessed for the presence of inducible perfusion defects (either regional or circumferential hypoperfusion) and late-gadolinium enhancement (LGE). If there was disagreement between observers, a third experienced reporter's opinion was sought. Heart rate response to vasodilator (assessed as adequate if increase of ≥ 10 beats per minute) and the identification of splenic switch-off sign (adenosine only) were used to evaluate the hyperemic stress response [25]. If at least one of these criteria were fulfilled, the patient was included in the analysis.

Perfusion maps were analyzed using MATLAB (Mathworks Inc, Natick, Massachusetts), with segmentation of the myocardium based on the American Heart Association (AHA) 17-segment myocardial model (the apical cap was not imaged). Analysis consisted of manual delineation of the endocardial and epicardial contours, with identification of the superior right ventricular insertion point on a separate map to avoid analysis bias. Following this, 600 sampling areas were automatically generated within each slice (60 circumferential points within 10 transmural layers) [26]. Global MBF was calculated as the mean of MBF values from all 1800 sampling points. Global myocardial perfusion reserve (MPR) was calculated as the ratio of global stress MBF and global rest MBF. Utilizing the high spatial resolution of the automated quantitative perfusion mapping, analysis of the global endocardial and epicardial MBF was conducted in all patients. The endocardial MBF represented the mean MBF values of the inner half of the LV wall, whilst the epicardial MBF was calculated as the mean MBF values of the outer half of the LV wall. Mean MBF was used to calculate coronary vascular resistance (CVR, defined as MAP at stress or rest divided by corresponding MBF).

### 2.5 Data analysis

Calculation of hyperemic stress MBF was conducted in all analyzed patients. MPR and rest MBF analysis were only conducted in patients receiving adenosine and only in those who received rest perfusion imaging. Specific sub-analysis to correct for various co-factors was conducted. In analysis where LGE was excluded, all those patients with any evidence of LGE within any section of the myocardium as identified by the accredited physicians were removed from the analysis for those specific calculations. Similarly, in controlling for potential effects of left ventricular systolic impairment, only those patients with preserved left ventricular ejection fraction (LVEF ≥ 55%) were included within that specified aspect of sub-analysis. A further sub-analysis was performed by splitting the cohort into distinct age categories: less than 55 years old (young adults); 55–74 years old (middle-aged); and 75 years old or greater (elderly).

### 2.6 Statistical analysis

Statistical analysis was conducted using SPSS Statistics version 29 (IBM, Armonk, New York). Patients receiving regadenoson, or those without rest MBF measurements, were excluded from analysis of all variables influenced by rest MBF (e.g., MPR). Data was assessed for normality by Shapiro–Wilk testing, but variables were not normally distributed. As such, nearly all continuous variables were reported as median value with interquartile range (IQR); categorical variables are represented as frequencies with corresponding percentage. The exception to this was adenosine dose comparison which was reported as mean dose ± standard deviation. Statistical significance between two categorical groups was established using the Mann–Whitney U test; when assessing more than two categorical groups, Kruskal–Wallis testing was utilized. Significant differences between paired variables (e.g., endocardial and epicardial MBF values) were established using the Wilcoxon signed-rank test. Univariate (via Pearson coefficient) and multivariate linear regression analyses were conducted to assess the relationship of potential variables contributing to stress MBF and MPR values. When assessing perfusion values categorized by sex, females were coded as ‘1’; males as ‘2’ – negative correlation coefficients suggested lower values in men, whilst positive coefficients suggested lower values in women. Significance was evaluated using 2-sided testing; a p value < 0.050 was considered statistically significant. All graphs and plots were generated using Matplotlib version 3.7.1 via Python 3 (https://doi.org/10.5281/zenodo.7697899, 2023) [27].

## 3 Results

**Fig. 2:**
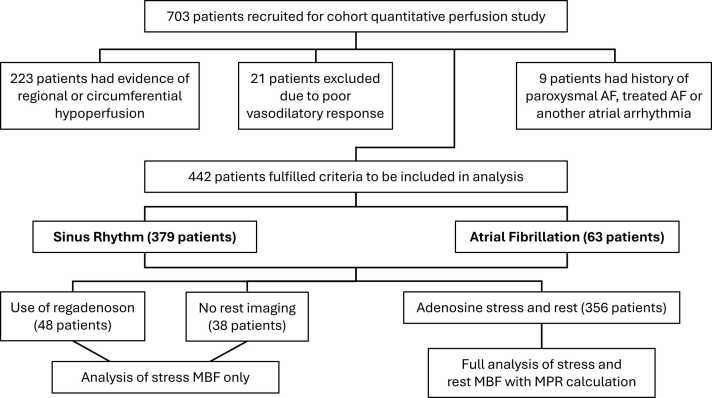
Patient flow chart demonstrating how patients were selected for study analysis. Eligible patients were categorized as either sinus rhythm or atrial fibrillation with comparative analysis of stress MBF in all participants, and rest MBF and MPR in only those undergoing both rest and adenosine stress imaging. *MBF* myocardial blood flow, *MPR* myocardial perfusion reserve

Seven-hundred and three patients were recruited. Automated quantitative maps were not generated in eight patients, due to contrast administration errors during acquisition of the research sequence. 223 scans had inducible regional or circumferential perfusion defects identified and 9 patients had a history of paroxysmal AF, treated AF or other significant atrial arrhythmia and were excluded from analysis. Additionally, 21 patients were deemed to have poor vasodilator response and were also excluded. Fig. 2.

A total of 442 patient scans were analyzed further. Exactly 63 of these patients were identified for the AF subgroup, while the remaining 379 patients were in sinus rhythm. Splenic switch off was identified at hyperemia in all analyzed patients receiving adenosine. Rest MBF and MPR were not analyzed in 86 patients due to either the use of regadenoson as the vasodilatory agent (48 patients) or rest imaging not being performed in those receiving adenosine (38 patients). Out of the 356 patients where rest MBF and MPR were analyzed, there were 50 in the AF subgroup and 306 in the sinus rhythm subgroup.

The baseline characteristics for all analyzed patients are shown in Table 1. Patients in AF were older and more likely to be male. Use of beta-blockers was more prevalent in those with AF, likely associated with both the underlying arrhythmia and lower median LVEF. Patients in the AF subgroup were more likely to have ventricular myocardial scarring/fibrosis, and a similar distribution of risk factors for CAD was seen between the two subgroups.

**Table 1:** Baseline characteristics for all analyzed patients.

		Sinus Rhythm (*N = 379)*	Atrial Fibrillation *(N = 63)*	Significance *(2-sided p)*
Age (years)		60 [51–67]	70 [62–74]	*<0.001*
Sex	*Male*	234 [61.7]	49 [77.8]	*0.014*
Diabetes mellitus		77 [20.3]	12 [19.0]	0.816
Hypercholesterolemia		199 [52.5]	30 [47.6]	0.472
Hypertension		196 [51.7]	38 [60.3]	0.205
Smoking history	*None*	201 [53.0]	30 [47.6]	0.337
	*Ex-smoker*	134 [35.4]	28 [44.4]	
	*Current smoker*	44 [11.6]	5 [7.9]	
Use of beta-blockers		158 [41.7]	50 [79.4]	*<0.001*
History of CAD		137 [36.1]	17 [27.0]	0.157
Previous coronary intervention		75 [19.8]	7 [11.1]	0.209
Stress agent	*Adenosine* *Regadenoson*	341 [90.0]	53 [84.1]	0.167
		38 [10.0]	10 [15.9]	
LV ejection fraction (%)		59 [52–63]	46 [39–52]	*<0.001*
Indexed LV end-diastolic volume (ml/m²)		80.7 [68.9–95.6]	79.6 [70.3–97.2]	0.932
Indexed LV myocardial mass (g/m²)		52.0 [44.7–62.9]	57.1 [47.0–68.8]	*0.023*
Presence of LGE	*None*	233 [61.5]	23 [36.5]	*0.003*
	*Ischemic*	77 [20.3]	18 [28.6]	
	*Non-ischemic*	62 [16.4]	21 [33.3]	
	*Mixed*	7 [1.8]	1 [1.5]	

The most significant differences between the two groups were mean age, mean LV ejection fraction, frequency of beta-blocker use, and presence of LGE

LV end-diastolic volume and myocardial mass indexed to body surface area (Mosteller method). *CAD* coronary artery disease, *LV* left ventricle, *LGE* late-gadolinium enhancement

### 3.1 Haemodynamic measurements and effects of hyperemia

The baseline and hyperemic HR and BP measurements, and derived calculations, are shown in Table 2. The haemodynamic measurements, apart from systolic BP, differed between the two groups at rest, but no significant differences were seen during hyperemic stress. Despite a higher mean maximum adenosine dose in patients with AF compared with those in sinus rhythm (167.7 ± 29.4 μg/kg/min vs. 146.2 ± 16.9 μg/kg/min, p<0.001), the resultant increase in HR was significantly lower in the AF group. There was no significant difference in the median change (stress minus rest) of both systolic and diastolic BP between the two groups.

**Table 2:** A comparison of haemodynamic measurements between patients in sinus rhythm and AF.

	Sinus Rhythm (*N = 379)*	Atrial Fibrillation *(N = 63)*	Significance *(2-sided p)*
Rest			
*Heart rate (bpm)*	65 [58–75]	72 [62–80]	*0.002*
*Systolic BP (mmHg)*	127 [115–143]	135 [117–152]	0.112
*Diastolic BP (mmHg)*	76 [68–84]	80 [73–89]	*0.013*
*MAP (mmHg)*	93 [85–104]	100 [87–108]	*0.028*
*RPP (mmHg bpm)*	8385 [7112– 10,080]	9548 [8208– 10,640]	*<0.001*
Stress			
*Heart rate (bpm)*	89 [78–100]	88 [77–100]	0.477
*Systolic BP (mmHg)*	129 [118–143]	135 [118–155]	0.086
*Diastolic BP (mmHg)*	74 [67–84]	77 [69–87]	0.134
*MAP (mmHg)*	92 [85–102]	99 [85–108]	0.106
*RPP (mmHg bpm)*	11,550 [9600– 13,699]	12,091 [9660– 13,692]	0.560
Change (Stress minus Rest)			
*Heart rate (bpm)*	21 [16–29]	15 [10–21]	*<0.001*
*Systolic BP (mmHg)*	1 [–6–8]	3 [–8–13]	0.592
*Diastolic BP (mmHg)*	–1 [–6–5]	–2 [–8–3]	0.322

Median haemodynamic measurements were all increased at rest in the AF group. However, the median increase in heart rate during stress was significantly lower in patients with AF compared with sinus rhythm

Rest measurements were acquired for all patients prior to vasodilator administration. *BP* blood pressure, *bpm* beats per minute, *MAP* mean arterial pressure, *RPP* rate-pressure product

### 3.2 Comparison of myocardial blood flow

**Fig. 3:**
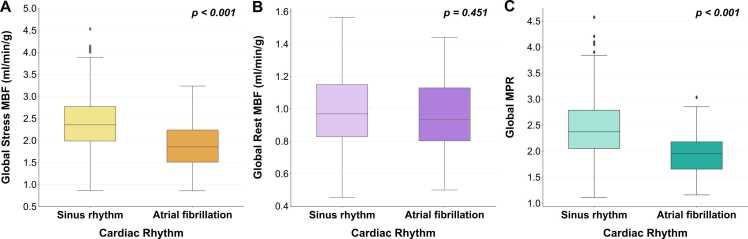
Comparison of perfusion values in patients in sinus rhythm compared with patients in AF. Both stress MBF [A] and MPR [C] are reduced in patients with AF (both p<0.001), whilst there is no significant difference in rest MBF [B] between the two groups (p = 0.451). *AF* atrial fibrillation, *MBF* myocardial blood flow, *MPR* myocardial perfusion reserve

Global stress MBF was significantly lower in patients with AF compared with those in sinus rhythm (median stress MBF 1.85 [1.52–2.24] mL/min/g vs. 2.35 [1.98–2.77] mL/min/g, p<0.001 [Fig. 3]). In patients with AF, there was no significant difference in stress MBF between the vasodilatory stress agent used (median stress MBF: adenosine 1.87 [1.50–2.24] mL/min/g; regadenoson 1.80 [1.50–2.20] mL/min/g; p = 0.686). Global MPR was also reduced in the AF group compared with sinus rhythm (median MPR 1.95 [1.62–2.19] vs. 2.37 [2.05–2.80], p<0.001). However, there was no significant difference between the two groups with regards to rest MBF (median rest MBF 0.93 [0.80–1.13] mL/min/g vs. 0.97 [0.83–1.15] mL/min/g, p = 0.451).

During hyperemia, CVR was significantly higher in patients with AF compared with sinus rhythm (51.1 [42.1–62.4] mHg/(mL/min/g) vs. 40.0 [33.2–48.1] mmHg/(mL/min/g), p<0.001). Additionally, there was a minimal but significant difference in CVR at rest between the two groups (AF: 99.8 [86.9–125.6] mmHg/(mL/min/g) vs. sinus rhythm: 94.2 [81.7–113.1] mmHg/(mL/min/g), p = 0.048).

### 3.3 Effects of physiological/pathological covariates and confounders on MBF

**Fig. 4:**
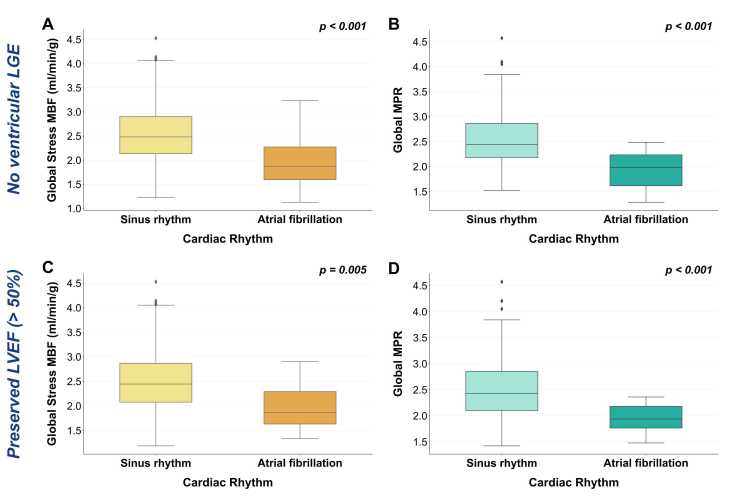
Comparison of perfusion values in subgroups of patients with no evidence of ventricular enhancement on LGE imaging [A & B] and preserved left ventricular ejection fraction (LVEF > 50%) [C & D]. Stress MBF and MPR were reduced in patients with AF in both subgroups. *LGE* late-gadolinium enhancement, *AF* atrial fibrillation, *MBF* myocardial blood flow, *MPR* myocardial perfusion reserve, *LVEF* left ventricular ejection fraction

Due to reduced MBF in areas of scarred myocardium, the presence of late-gadolinium enhancement was considered a confounder to MBF calculation [28]. A sub-analysis of patients without evidence of LGE demonstrated that, similar to the results for the entire cohort, both stress MBF and MPR were significantly reduced in the presence of AF compared with sinus rhythm (median stress MBF 1.87 [1.58–2.35] mL/min/g vs. 2.48 [2.13–2.91] mL/min/g, p<0.001; median MPR 1.98 [1.57–2.24] vs. 2.44 [2.18–2.87], p<0.001 [Fig. 4, top row]). Rest MBF in the absence of LGE was similar for both AF and sinus rhythm (0.94 [0.81–1.14] mL/min/g vs. 0.98 [0.84–1.21] mL/min/g, p = 0.587).

Analysis was also conducted to investigate the effects of potential covariates. In patients with preserved LVEF, stress MBF and MPR were reduced in the presence of AF (median stress MBF 1.87 [1.52–2.35] ml/min/g vs. 2.45 [2.08–2.87] ml/min/g, p = 0.005; median MPR 1.94 [1.70–2.26] vs. 2.43 [2.10–2.85], p<0.001 [Fig. 4, bottom row]). Again, there was no significant difference between rest MBF in AF or sinus rhythm in those with preserved LVEF (median rest MBF 1.08 [0.85–1.21] mL/min/g vs. 0.98 [0.83–1.18] mL/min/g, p = 0.602).

**Fig. 5:**
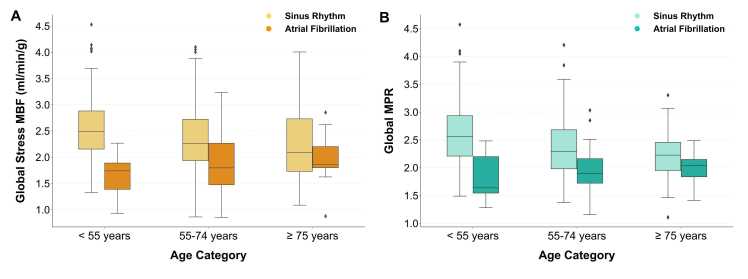
Sub-analysis of global perfusion values in both AF and sinus rhythm across age categories. Stress MBF and MPR are reduced in both the <55 years and 55–74 years categories (all p<0.001), but no difference is seen in the ≥75 years category for either stress MBF (p = 0.346) and MPR (p = 0.149). *AF* atrial fibrillation, *MBF* myocardial blood flow, *MPR* myocardial perfusion reserve

The effects of age on MBF were also considered by dividing the cohort into three discrete age categories and comparing AF and sinus rhythm (< 55 years: n=139, AF=6.5%; 55–74 years: n=256, AF=15.6%; ≥ 75 years: n=47, AF=29.8%). In both the < 55 years and 55–74 years categories, stress MBF and MPR were significantly reduced in AF compared with sinus rhythm (all p<0.001) [Fig. 5]. However, in the ≥ 75 years category, no significant difference was seen between the two groups (stress MBF: p = 0.346; MPR: p = 0.149). No significant differences in rest MBF were seen between AF and sinus rhythm in any age category. Analysis of MBF across age categories demonstrated a reduction in both stress MBF and MPR with age for those in sinus rhythm (both p < 0.001). However, there was no significant difference in MBF values across the age categories in the presence of AF (stress MBF: p = 0.179; MPR: p = 0.644).

Multivariate linear regression analysis was performed to calculate the true effects of covariates on MBF. This analysis excluded participants with evidence of LGE (as this was considered a confounder). Age, baseline LVEF, and the presence of AF during CMR were assessed, alongside other demographic differences between the two groups (indexed LV myocardial mass, sex, and the use of beta-blockers at the time of the scan) [Table 3]. The presence of AF was an independent factor contributing to stress MBF (p = 0.007) and MPR (p<0.001). Participant sex was the only other factor to demonstrate a significant relationship with both stress MBF and MPR. The correlation values were negative for stress MBF, suggesting that women have a higher stress MBF than men, whilst the correlation values are positive for MPR, suggesting the opposite relationship with regards to MPR. Indexed LV myocardial mass demonstrated a contributing relationship to MPR, but not to stress MBF, whilst the use of beta-blockers was an independent contributing factor to stress MBF but not MPR. Age and LVEF did not significantly contribute to either perfusion measurement.

**Table 3:** Comparison of univariate and multivariate linear analysis for factors affecting MBF.

	Univariate Analysis		Multivariate Linear Analysis		
	R	p-value	B	ß	p-value
Stress MBF (N = 256)					
*Age*	−0.128	*0.041*	−0.004	−0.076	0.206
*Sex*	−0.352	*<0.001*	−0.413	−0.320	*<0.001*
*Presence of AF*	−0.258	*<0.001*	−0.384	−0.171	*0.007*
*Use of beta-blockers*	−0.201	*0.001*	−0.184	−0.136	*0.028*
*LVEF*	0.154	*0.014*	−0.001	−0.014	0.828
*Indexed myocardial mass*	−0.235	*<0.001*	−0.003	−0.051	0.444
MPR (N = 206)					
*Age*	−0.185	*0.008*	−0.005	−0.121	0.081
*Sex*	0.119	0.090	0.237	0.215	*0.005*
*Presence of AF*	−0.317	*<0.001*	−0.579	−0.320	*<0.001*
*Use of beta-blockers*	−0.057	0.413	0.082	0.073	0.308
*LVEF*	0.076	0.275	−0.001	−0.012	0.870
*Indexed myocardial mass*	−0.056	0.420	−0.007	−0.160	*0.039*

p < 0.050 displayed in italics, denoting significance. R = Pearson coefficient; B = unstandardized coefficient; ß = standardized coefficient. *MBF* myocardial blood flow, *MPR* myocardial perfusion reserve, *AF* atrial fibrillation, *HR* heart rate, *LVEF* left ventricular ejection fraction

Participant sex and presence of AF are independent factors contributing to both stress MBF and MPR values

### 3.4 Endocardial and epicardial MBF assessment

**Fig. 6:**
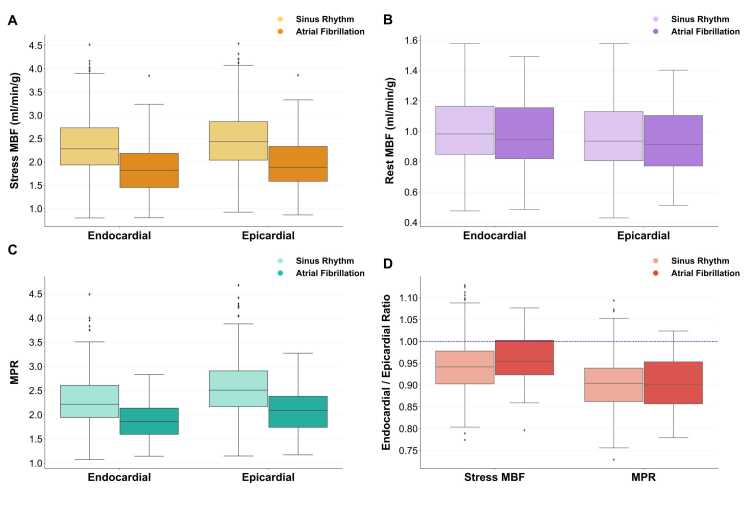
Analysis of endocardial and epicardial perfusion values in both AF and sinus rhythm. Both endocardial and epicardial stress MBF [A] and MPR [C] are significantly lower in AF (p < 0.001 for all). Endocardial stress MBF and MPR were reduced compared to paired epicardial values, and the endocardial/epicardial ratios for stress MBF and MPR were all below 1 [D]. *AF* atrial fibrillation, *MBF* myocardial blood flow, *MPR* myocardial perfusion reserve

Both endocardial and epicardial stress MBF and MPR were lower in AF than in sinus rhythm (all p<0.001) [Fig. 6]. Additionally, endocardial stress MBF and MPR were significantly reduced compared to the paired epicardial values (all p<0.001), regardless of whether the patients were in sinus rhythm or AF. As such, the majority of patients had endocardial/epicardial ratios for both stress MBF and MPR below 1 regardless of underlying rhythm [Fig. 6D]. With regards to stress MBF, the endocardial/epicardial ratio was higher in AF than in sinus rhythm (median stress MBF ratio, AF 0.95 [0.92–1.00] vs. sinus rhythm 0.94 [0.90–0.98], p = 0.039), but no significant difference was seen in the ratio derived from endocardial and epicardial MPR (median MPR ratio, AF 0.90 [0.85–0.96] vs sinus rhythm 0.90 [0.86–0.94], p = 0.694). There was no significant difference between AF and sinus rhythm with regards to endocardial and epicardial MBF at rest (endocardial p = 0.507; epicardial p = 0.297), but in both groups, endocardial MBF was higher than epicardial MBF at rest (both p<0.001).

## 4 Discussion

This study demonstrates that MBF assessment can be achieved in patients with AF using a high-resolution free-breathing perfusion CMR sequence with automated motion-compensation and quantitative perfusion mapping. Quantitative perfusion mapping potentially offers novel insights into the pathophysiology of AF.

MBF during hyperemic stress was demonstrated to be significantly lower in the presence of atrial fibrillation during the scan compared with sinus rhythm, contributing to an associated reduction in MPR values. Only one other study within stress perfusion CMR has previously analyzed quantitative rest and hyperemic stress MBF measurements in patients with AF, similarly demonstrating reduced stress MBF in the presence of AF compared with sinus rhythm [6]. Furthermore, these findings are comparable to those seen in similar studies performed with other non-invasive imaging modalities such as computed tomography (CT) and positron-emission tomography (PET) [7], [8], [9], [10]. Moreover, by utilizing the high-resolution of this stress perfusion CMR sequence, this study is the first to observe that both endocardial and epicardial MBF are reduced in AF compared with sinus rhythm.

The difference in stress MBF and MPR between the two groups was maintained despite controlling for suspected confounders (presence of ventricular LGE) and covariates (LVEF and age). In addition, multivariate linear regression analysis demonstrated that the presence of AF during the scan was a significant, independent factor contributing to the lower stress MPF and MPR seen in this group.

### 4.1 Haemodynamic considerations affecting MBF and MPR in AF

The haemodynamic measurements at baseline were significantly different between the two groups. Despite a higher proportion of patients taking beta-blockers, the resting HR and both systolic and diastolic BP were higher in the AF group. Consequently, the RPP was significantly higher in AF patients at rest. Similar findings have been described in other studies, regardless of the proportion of patients taking beta-blockers [6], [7], [8], [9]. Although the HR seen at peak hyperemia is similar between the two groups, the overall increase in HR is lower in AF patients despite a higher proportion of patients requiring increasing doses of adenosine. This is unlikely to simply be a factor of increased beta-blockade in the AF group – a study conducted using cardiac PET found a similar diminished HR response despite the proportion of beta-blockade in both AF and sinus rhythm groups being in excess of 90% [7]. Importantly though, the degree of BP change between stress and rest was the same in both groups, suggesting that both groups of patients were sufficiently stressed. Indeed, splenic switch-off was observed in all analyzed patients achieving hyperemia via adenosine, indicative that the difference in HR between the two groups was not attributed to underestimation of the required adenosine dose in the AF group. Additionally, the analysis demonstrated no significant difference in stress MBF between adenosine and regadenoson for patients in AF.

Overall, these findings suggest that the HR response to intravenous vasodilator is inherently reduced in AF despite an adequate degree of vasodilator administration. With no change in the rest MBF between AF and sinus rhythm, it can be assumed that the difference in stress MBF response between the groups was the sole contributor to the change in MPR. This may be associated with the significant increase in CVR observed in AF patients during hyperemia, which was similarly seen in other studies [8], [9].

### 4.2 The effects of covariates on hyperemic MBF and MPR

Although it was demonstrated that AF was an independent factor affecting MBF, the multivariate linear regression analysis in this study also highlighted how strong other potential covariates may be in contributing to stress MBF and MPR values. Whilst previous research has suggested that reduced LVEF may contribute to lower stress MBF values, the effect of LVEF on both stress MBF and MPR in this study population was negligible [29]. Patient sex was the only other variable found to be associated with both stress MBF and MPR—the analysis suggested that stress MBF was reduced in men, while conversely MPR was increased in males compared with females. The effects of both sex and age on MBF have been studied previously, with comparable results seen [30]. The difference in stress MBF and MPR between AF and sinus rhythm was most profound in the youngest age category, suggesting that AF may have a greater effect on MBF and MPR in younger patients, although other variables such as higher prevalence of concomitant vasodilatory medication in the older age categories may be contributing to this effect. Further investigation into the interaction of the multiple co-variables affecting MBF during hyperemia is warranted.

### 4.3 Transmural perfusion distribution

In the absence of proven CAD, it has been suggested that coronary microvascular dysfunction (MVD) is a major contributing factor to the reduced MBF observed in patients with AF during hyperemic stress [11]. Indeed, computational fluid dynamics analysis previously demonstrated reduced microcirculatory myocardial blood flow in AF compared with sinus rhythm, particularly at high ventricular rates when beat-to-beat relative differences in cardiac output are more pronounced [31]. However, in patients with chronic AF (persistent/permanent) the effects of medication and/or metabolic autoregulation are less well understood.

Analysis of endocardial/epicardial MBF and MPR ratios has previously been used to assess microvascular blood flow in quantitative perfusion CMR [32]. This study demonstrated that both endocardial stress MBF and MPR were significantly reduced compared with epicardial stress MBF and MPR, both in patients with AF and sinus rhythm. Conversely, endocardial MBF at rest was increased compared with epicardial MBF. This is in keeping with findings seen previously in healthy volunteers and likely a reflection of the higher metabolic activity in the subendocardium at rest [33]. Despite the reduction in stress MBF and MPR values in both the endocardium and epicardium of those in AF, the endocardial/epicardial difference appears to be proportionate compared with sinus rhythm. Whilst MVD may be one factor affecting hyperemic perfusion in AF, a global endocardial/epicardial ratio may not be sensitive to assess this. Likewise, other physiological mechanisms may be contributing to why no change in the endocardial/epicardial ratio was seen in AF.

## 5 Limitations

One limitation of this study is the discrepancy in the group sizes, with the AF group being significantly smaller than the sinus rhythm group. Although the groups were well balanced in terms of cardiovascular risk factors, there were some demographic differences between the groups, such as patient sex.

The identification of AF was conducted using the in-scanner ECG monitoring rather than 12-lead ECG assessment. This interpretation was performed by the supervising clinician, and it is possible that cardiac rhythm assessment may have been affected by either magnetohydrodynamic effects of the scanner, or human error resulting in misinterpretation.

The use of beta-blockers within the AF patient group was higher, whilst information about other concomitant therapeutic agents was not acquired. It is a logical assumption that the AF group may also have a higher prevalence of other vasodilatory/chronotropic medication, which may also contribute to changes in MBF. As all scans were performed as part of routine clinical investigations, medications were not withdrawn before scanning to ensure that conclusions from the stress perfusion CMR were representative of the patient’s normal haemodynamic status.

The use of RPP to correct the rest MBF and MPR values was not applied within this study. It has been suggested that not applying this correction could potentially lead to the observation of reduced MPR in patients with hypertension, leading to the overestimation of ischemic burden in these patients [34]. The prevalence of hypertension was not statistically different between the two groups, nor was there a difference in rest MBF. Moreover, application of RPP correction to rest MBF values is not widely practiced in stress perfusion CMR, and automated MBF quantification frameworks do not routinely apply this correction to rest MBF maps.

## 6 Conclusions

This study demonstrates that high-resolution free-breathing perfusion CMR with automated in-line quantitative mapping can be used to assess MBF in patients with AF. In the absence of CAD, patients in AF undergoing high-resolution free-breathing stress perfusion CMR with automated quantitative perfusion mapping had a reduced MBF during hyperemia and lower MPR compared with those in sinus rhythm, independent of other co-variables.

## Funding

The authors acknowledge financial support from the National Institute for Health Research (NIHR) Biomedical Research Centre award to Guy’s & St Thomas’ NHS Foundation Trust in partnership with 10.13039/100009360King’s College London, and the NIHR MedTech Cooperative for Cardiovascular Disease at Guy’s and St Thomas’ NHS Foundation Trust. Support was also received through the Wellcome Trust Innovator Award [222678/Z/21/Z] and the British Heart Foundation (BHF) Translational Award [BHF TG/18/2/33768]. Sven Plein is funded by a BHF Foundation Chair Award [CH/16/2/32089]. The views expressed are those of the authors and not necessarily those of the BHF, NHS, NIHR, or Wellcome Trust.

## Author contributions

**Richard J. Crawley:** Writing – review & editing, Writing – original draft, Visualization, Software, Resources, Project administration, Methodology, Investigation, Formal analysis, Data curation, Conceptualization. **Karl-Philipp Kunze:** Writing – review & editing, Writing – original draft, Supervision, Software, Methodology. **Anmol Kaushal:** Writing – review & editing, Writing – original draft. **Xenios Milidonis:** Writing – review & editing, Writing – original draft, Supervision, Software, Methodology, Investigation, Conceptualization. **Jack Highton:** Writing – review & editing, Software, Resources. **Blanca Domenech-Ximenos:** Writing – review & editing, Data curation. **Irum D. Kotadia:** Writing – review & editing, Methodology. **Can Karamanli:** Writing – review & editing, Software, Resources. **Nathan C.K. Wong:** Writing – review & editing, Software, Resources. **Robbie Murphy:** Writing – review & editing, Investigation, Data curation. **Ebraham Alskaf:** Writing – review & editing, Writing – original draft. **Radhouene Neji:** Writing – review & editing, Supervision, Software**. Mark O’Neill:** Writing – review & editing, Validation. **Steven E. Williams:** Writing – review & editing, Validation. **Cian M. Scannell:** Writing – review & editing, Writing – original draft, Supervision, Methodology, Investigation, Conceptualization. **Sven Plein:** Writing – review & editing, Writing – original draft, Validation, Supervision, Methodology. **Amedeo Chiribiri:** Writing – review & editing, Writing – original draft, Validation, Supervision, Resources, Project administration, Methodology, Funding acquisition, Conceptualization.

## Declaration of competing interests

The authors declare the following financial interests/personal relationships which may be considered as potential competing interests: Amedeo Chiribiri reports that financial support was provided by Wellcome Trust. Amedeo Chiribiri reports that financial support was provided by NIHR Biomedical Research Centre at Guy’s and St Thomas’ NHS Foundation Trust and King’s College London. Amedeo Chiribiri reports that financial support was provided by British Heart Foundation. Sven Plein reports a relationship with British Heart Foundation that includes funding grants. Karl-Philipp Kunze, Radhouene Neji have patent #Detection of Mis-Triggering in Heart MRI (EP4321893) pending to Siemens Healthcare GmbH. Karl-Philipp Kunze, Radhouene Neji have patent #PROSPECTIVE SLICE TRACKING THROUGH INTERLEAVED PERPENDICULAR ACQUISITIONS OF DYNAMIC 2D CARDIOVASCULAR MRI (EP4290262) pending to Siemens Healthcare GmbH. Karl-Philipp Kunze, Radhouene Neji have patent #COMPUTER-IMPLEMENTED METHOD, COMPUTER PROGRAM AND PROCESSING APPARATUS FOR RECONSTRUCTING A DYNAMIC SERIES OF MAGNETIC RESONANCE IMAGES (EP4290265) pending to Siemens Healthcare GmbH. If there are other authors, they declare that they have no known competing financial interests or personal relationships that could have appeared to influence the work reported in this paper.
